# Identification by Bioinformatics Analysis of Potential Key Genes Related to the Progression and Prognosis of Gastric Cancer

**DOI:** 10.3389/fonc.2022.881015

**Published:** 2022-05-04

**Authors:** Wencang Gao, Min Yang

**Affiliations:** Department of Oncology, The Second Affiliated Hospital of Zhejiang Chinese Medical University, Hangzhou, China

**Keywords:** gastric cancer, TNM stage, bioinformatics analysis, RNA-seq, tumor immune cell infiltration

## Abstract

**Objective:**

Despite increasingly sophisticated medical technology, the prognosis of patients with advanced gastric cancer is still not objectively certain. Therefore, it is urgent to identify new diagnostic and prognostic biomarkers. To identify potential critical genes related to gastric cancer’s staging mechanism and to the prognosis of gastric cancer.

**Methods:**

Dynamic trend analysis was conducted to find genes with similar trends in gastric cancer staging in order to explore the differentially expressed genes in gastric cancer and identify the intersection of the results of the dynamic trend analysis. Functional predictive analysis were performed on the obtained genes to observe the expression of prognostic genes in gastric cancer and in gastric cancer stages as well as the correlation with tumor immune cell infiltration. Gastric cancer samples were collected and sequenced for follow-up analysis based on the results of the Cancer Genome Atlas (TCGA) database analysis.

**Results:**

The expression of genes enriched in module 0 had a similar trend in gastric cancer staging. 3213 differential genes were screened. A total of 50 intersection genes were obtained among genes with similar trends, of which only 10 genes have prognostic significance in gastric cancer. These 10 genes were correlated with macrophage infiltration in varying degrees. In addition, we found that AGT was significantly abnormally expressed in the results of sample sequencing. AGT was related to the occurrence of gastric cancer and interacted with brd9, golph3, nom1, klhl25, and psmd11.

**Conclusion:**

AGT has prominent abnormal expression in gastric cancer and may promote gastric cancer progression. This study provides a new direction for further exploring potential biomarkers and molecular targeted gastric cancer therapy.

## Introduction

Gastric cancer is a common upper gastrointestinal cancer (1). It often occurs among the middle-aged and elderly and is not common in young people (under the age of 45), who represent no more than 10% of patients suffering from the disease (2). With improvements in treatment technology, the five-year survival rate of early gastric cancer can reach more than 95%, but the symptoms of gastric cancer are rare and nonspecific in the early stages of the disease (3). This multifactorial disease is associated with environmental and genetic factors and is usually diagnosed in its late stages, with a median survival period of less than 12 months (4, 5). Data show that the prognosis of patients is related to the tumor stage (6). Therefore, more and more attention has been paid to clinical staging in recent years. Obtaining accurate, reliable clinical staging is a challenging but crucial problem.

The Union for international cancer control (UICC) and the American Joint Committee on cancer (AJCC) maintain the TNM cancer control staging system, a tool used by physicians to stage different types of cancer (T), existence in the lymph nodes (N), and metastasis (M). At present, the TNM staging system is used to evaluate most malignant tumors worldwide, including gastric cancer (7). The accurate staging of gastric cancer is a crucial part of patient management, as tumor staging is the most important predictor of survival at the initial diagnosis. In addition, the treatment scheme for the tumor will vary according to different disease stages (8). Tumor prognosis prediction and treatment recommendations for gastric cancer are mainly guided by the TNM staging system (9). Preventable factors that play a role in gastric cancer development are also important. This study aimed to find biomarkers that may be involved in the progression and staging of gastric cancer to stratify patients and better target treatment.

## Methods and Analysis

### Data Collection

We extracted data from the Cancer Genome Atlas (TCGA)database, obtaining the mRNA information of gastric cancer samples, including 375 primary cancers and 32 non-tumor tissues. The original count data and relevant clinical information of patients were also downloaded and extracted. Gene expression information was matched to the clinical stage information, and unknown or incomplete clinical information was removed.

### Sample Collection

The gastric cancer tissues and adjacent tissues of five patients with gastric cancer in our hospital (two males and three females) were collected and preserved in liquid nitrogen. This study was approved by the Ethics Committee of the First Affiliated Hospital of Zhejiang University of Traditional Chinese Medicine. The duties, composition, operating procedures, and records of this ethics review committee follow the Measures for Ethical Review of Biomedical Research Involving Human Beings, the International Ethical Guidelines for Health-Related Research Involving Human Beings, the Declaration of Helsinki, international ethical guidelines such as GCP and ICH-GCP, and relevant domestic laws and regulations (2022-LW-015-01).

### Dynamic Trend Analysis

Trend analysis can classify genes with similar change characteristic patterns within a changing trend. We used the OmicShare online tool (https://www.omicshare.com/tools/) for analysis. *P* <.05 was considered statistically significant. The number of trends is chosen to be 20.

### Analysis of Differentially Expressed Genes

We used the limma package to identify differentially expressed genes between tumor tissues and normal tissues, followed by a test to analyze differentially expressed genes. Adj.*P* <.05 and | log2 (FC) | > 1.0 were used as cutoff values.

### Analysis of Tumor Immune Cell Infiltration

The correlation between gene expression and immune cells (including B cells, CD4 + T cells, CD8 + T cells, macrophages, neutrophils, and dendritic cells) was based on the TIMER database. *P* <.05 was considered statistically significant. For the spearman test, there is generally a significant correlation for |r|>0.95; a high correlation for |r|≥0.8; a moderate correlation for 0.5≤|r|<0.8; and a low correlation for 0.3≤|r|<0.5.

### Survival Analysis

Univariate Cox analysis was used to analyze the genes with prognostic characteristics in gastric cancer and to display the *p*-value, risk coefficient HR, and confidence interval. The KM curve was drawn with the “gene and survival” tool, which the R software package implements. *P* <.05 was considered statistically significant.

### Weighted Gene Coexpression Network Analysis

WGCNA has been widely used to identify disease-related gene modules and to extract potential therapeutic targets. First, the integral function in the WGCNA software package in R was used to select the appropriate soft threshold power β. We constructed a topological overlap matrix and hierarchical clustering tree between the module detected genes. Analysis and biological function analysis then further screened the key co expression modules. For visualization, the genes coexpressed with AGT were analyzed by Cytoscape software (https://cytoscape.org/).

## Results

### Dynamic Trend Analysis

**Figure 1:**
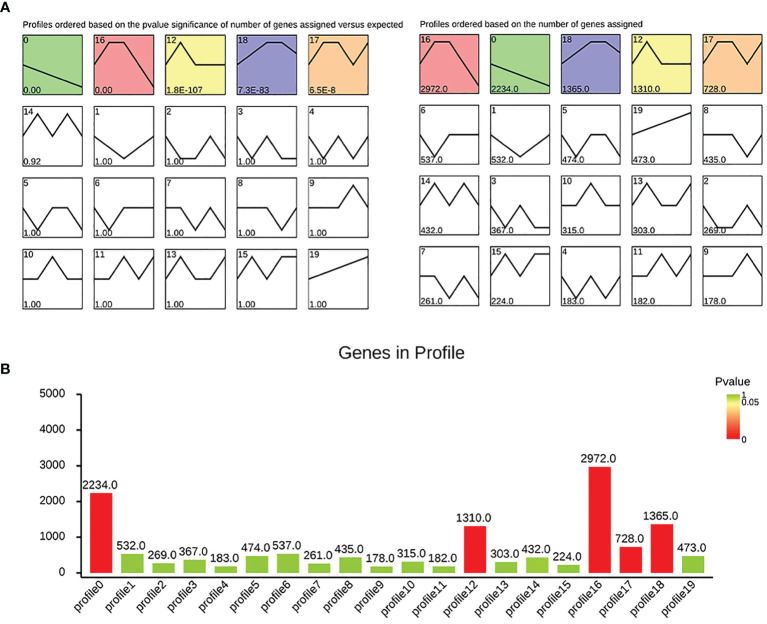
Dynamic trend analysis: **(A)** the significance and gene number of each module; **(B)** histogram showing the situation of each module.

First, we performed trend analysis based on TCGA to observe the dynamic expression of genes in different stages. Our analysis results show that the genes enriched in modules 0, 12, 16, 17, and 18 were statistically significant, and the genes enriched in module 0 show a downward linear trend. In module 0, 2,234 genes were enriched (see **Figure 1** for details).

### Screening of Differentially Expressed Genes

**Figure 2:**
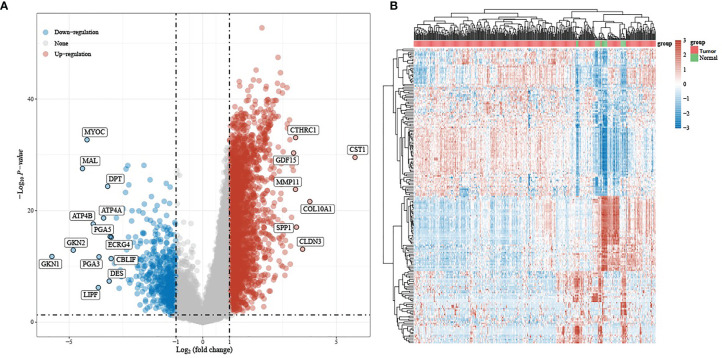
Differential expression analysis: **(A)** volcano plot of differentially expressed genes, with red indicating up-regulated genes and blue indicating down-regulated genes; **(B)** heat map of differential gene expression.

We then analyzed the differential expression between gastric cancer and normal tissues based on TCGA. Taking *P* <.05 and | log2 (FC) | > 1 as the screening thresholds, a total of 3,213 differential genes were displayed, of which 2,702 were up-regulated and 511 were down-regulated (see **Figure 2** for details).

### Intersecting Genes

**Figure 3:**
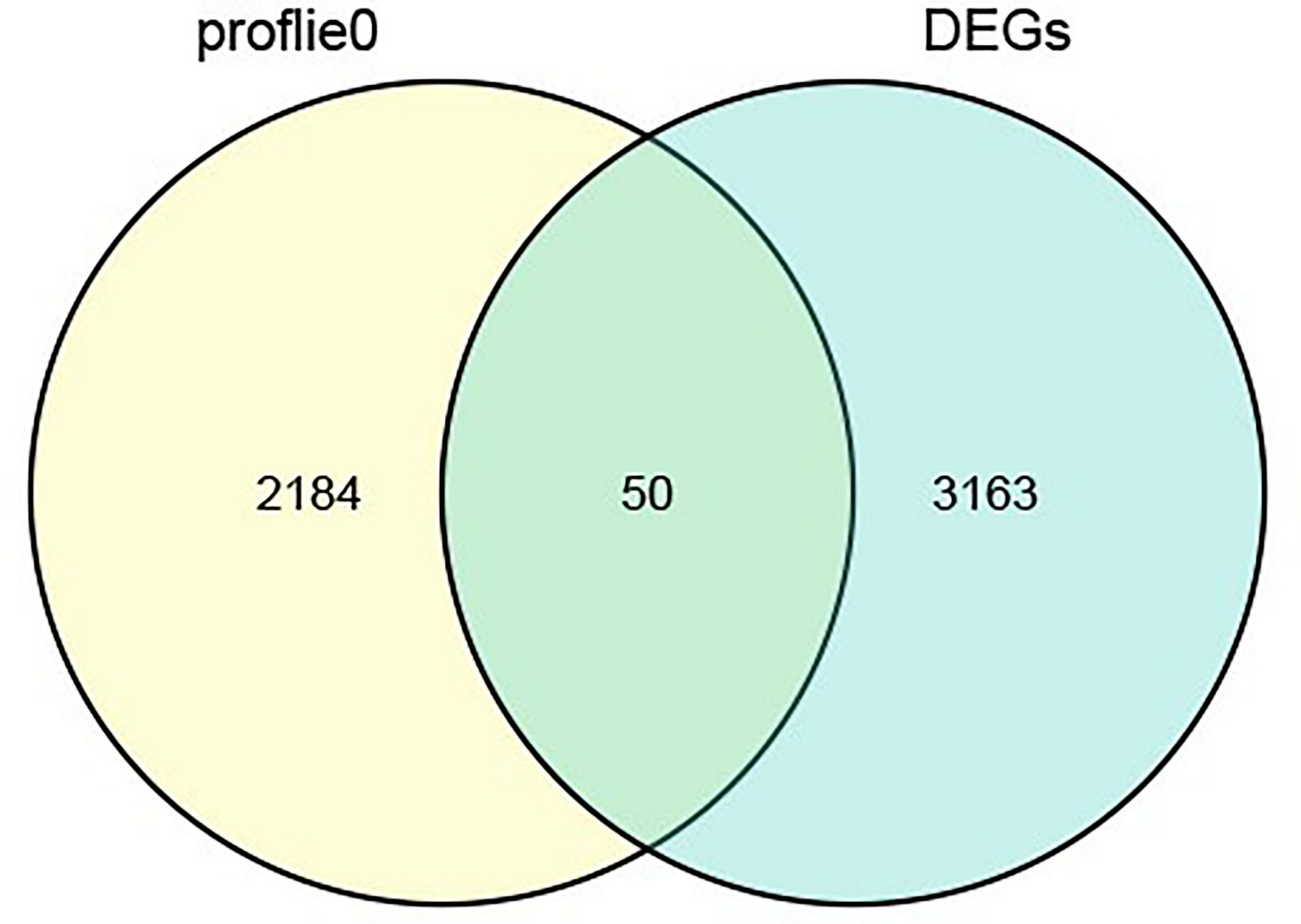
Venn diagram of the intersection.

Next, we intersected the genes of module 0 and the differential genes in the trend analysis results, yielding a total of 50 intersecting genes. (see **Figure 3** for details).

### Survival Analysis

**Figure 4:**
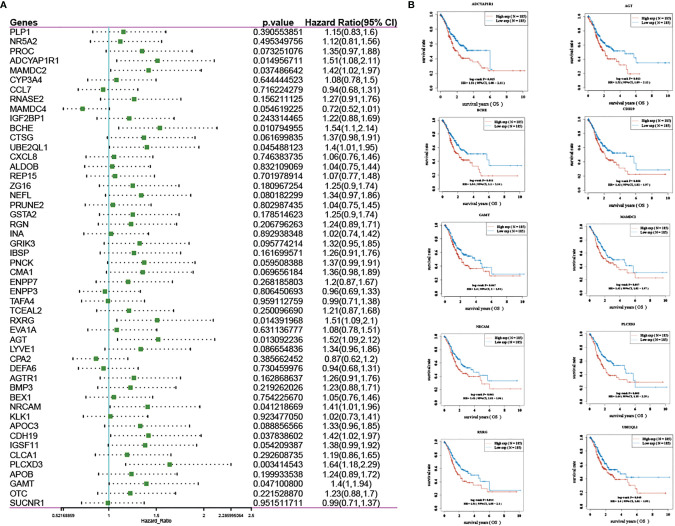
Prognosis analysis: **(A)** forest map of screening prognostic genes; **(B)** KM curve of genes with prognostic significance.

In addition, we analyzed the prognosis of the 50 intersecting genes and observed the effect of their expression on the prognosis of patients with gastric cancer. Ten of the 50 genes (RXRG, AGT, BCHE, UBE2QL1, PLCXD3, ADCYAP1R1, NRCAM, MAMDC2, CDH19, and GAMT) had prognostic significance in gastric cancer and were represented by KM curves (see **Figure 4**).

### Gene Expression in Gastric Cancer Tissues and Diverse Stages

**Figure 5:**
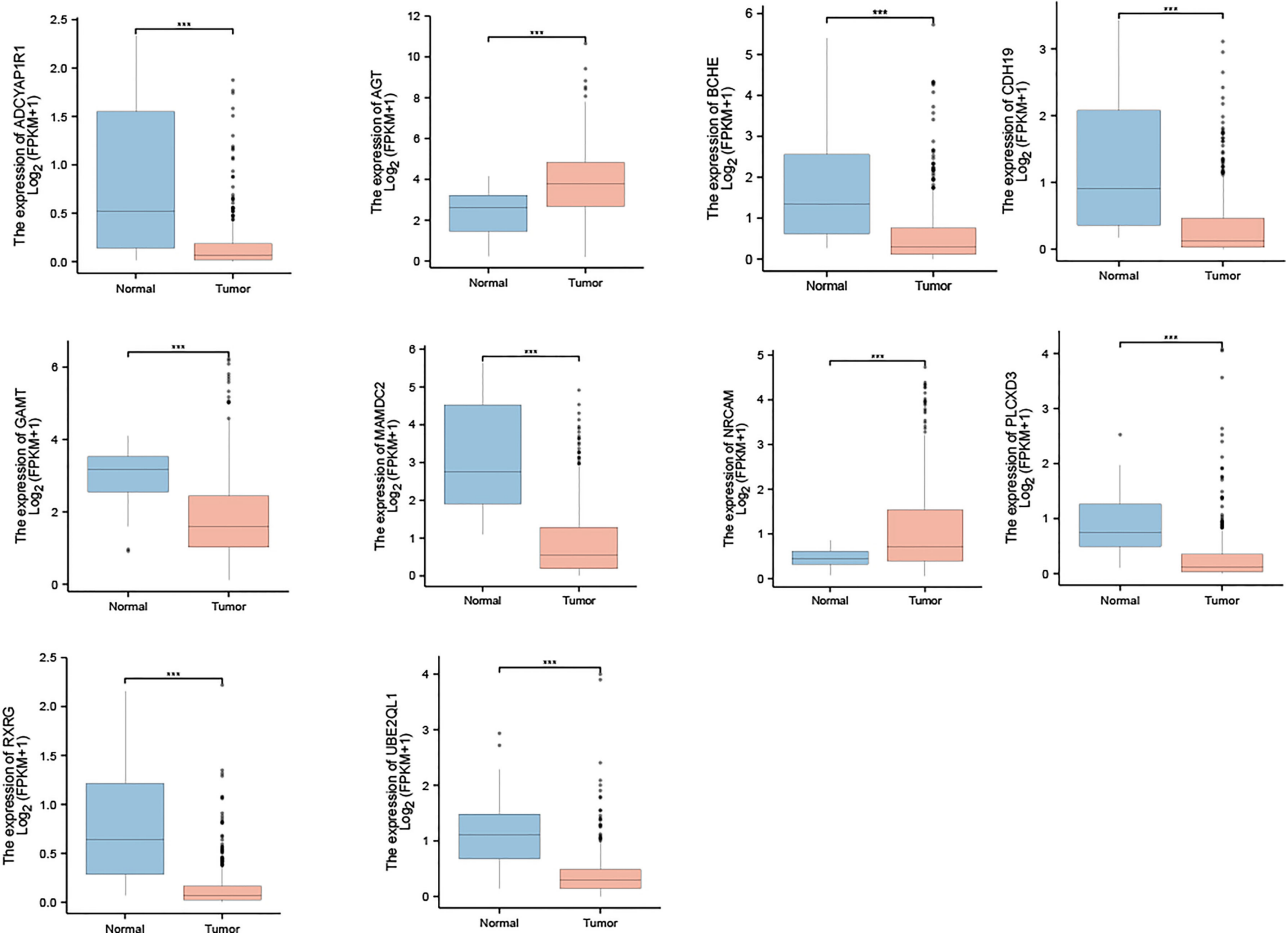
Expression of 10 genes in gastric cancer and normal tissues. ***p < 0.001.

**Figure 6:**
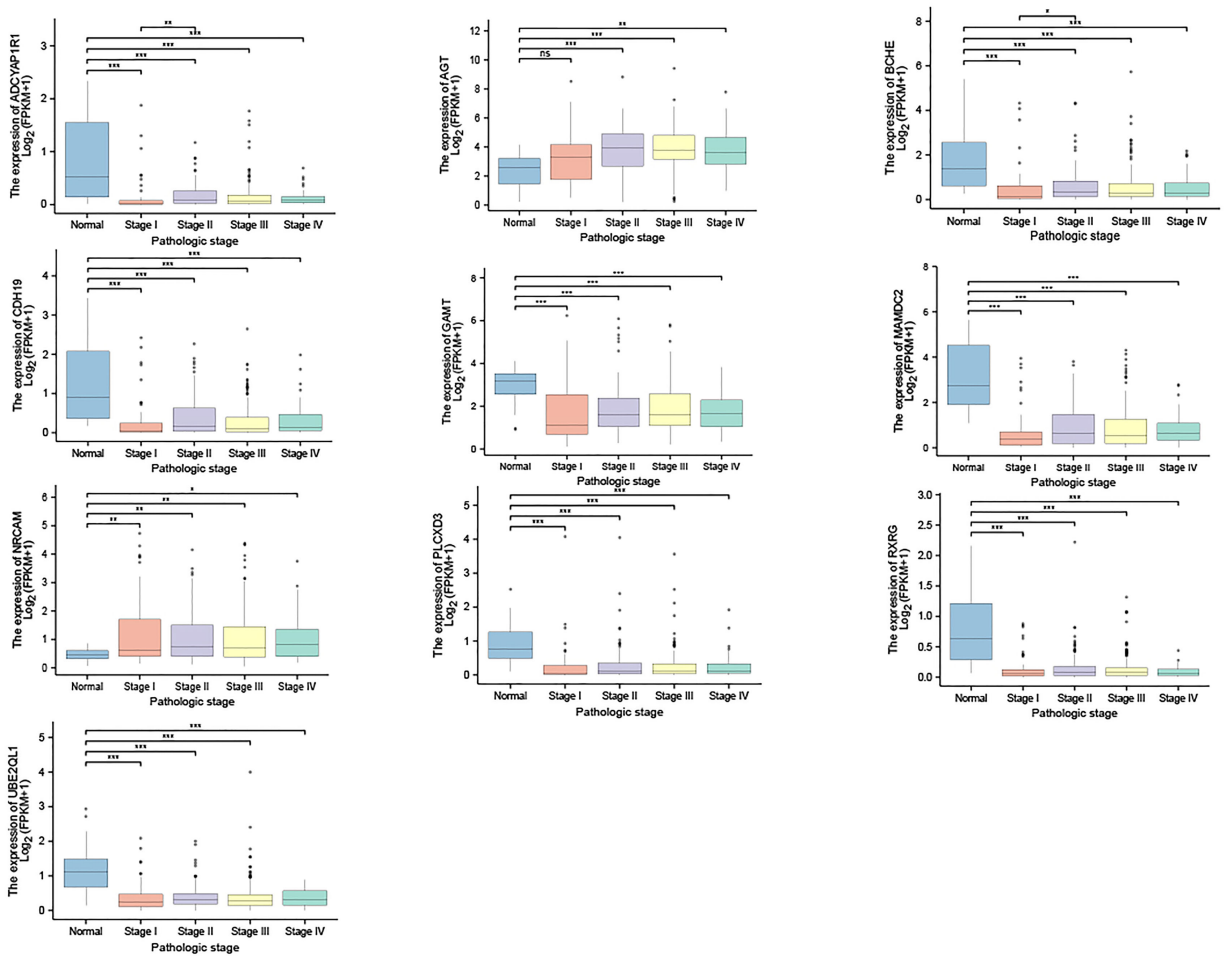
Expression of 10 genes in diverse stages of gastric cancer. *p < 0.05; **p < 0.01; ***p < 0.001; ns, non significant.

We then observed the expression of those 10 genes in gastric cancer and normal tissues. The results show that, compared with normal tissues, the expression of AGT and NrCAM in gastric cancer was significantly up-regulated while the expression of the other eight genes was down-regulated in gastric cancer (see **Figure 5** for details). In addition, we observed the expression of the 10 genes in various stages of gastric cancer, as shown in **Figure 6**.

### Relationship Between Gene Expression and Immune Cell Infiltration

**Figure 7:**
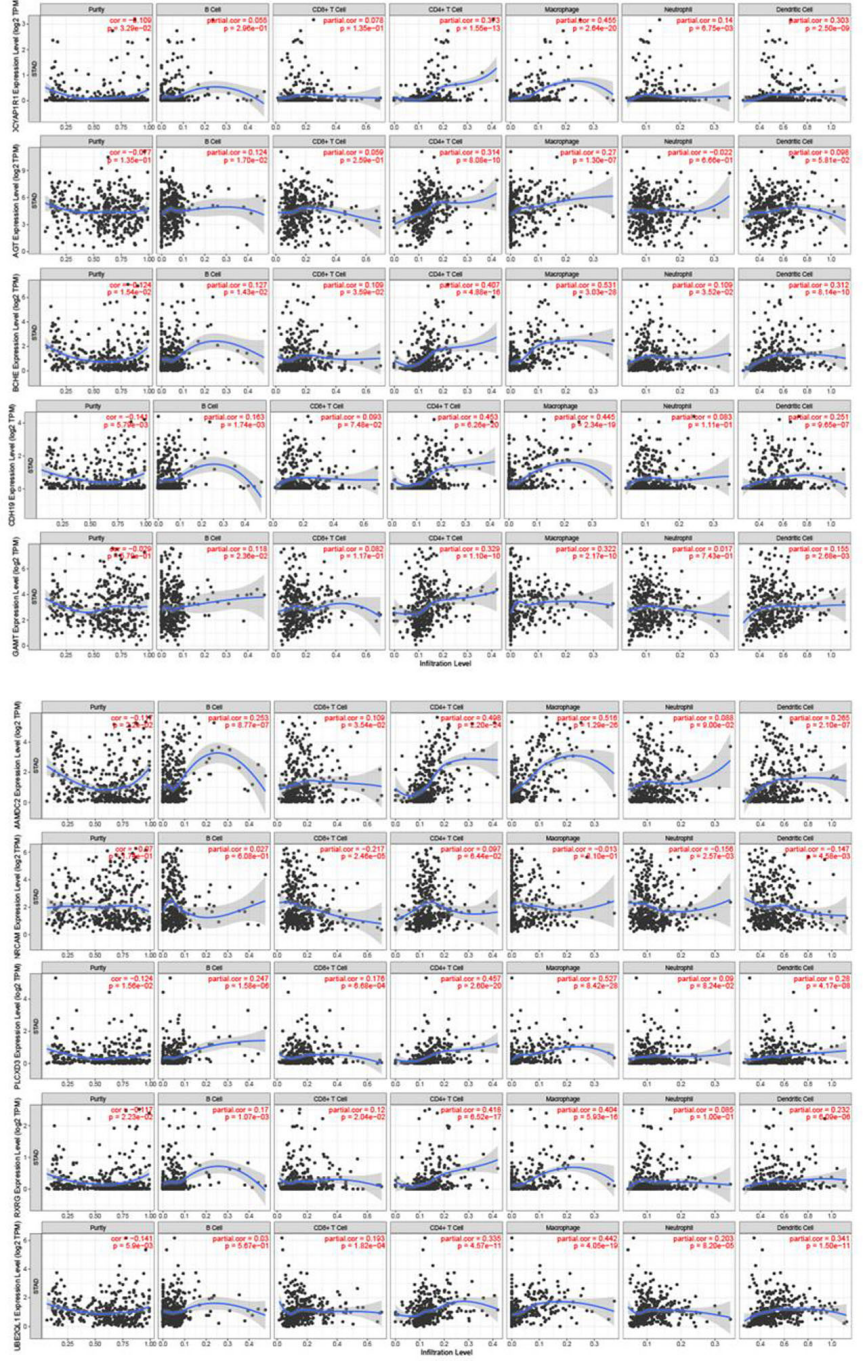
10 Relationship between gene expression and immune cells.

We further explored the relationship between those 10 genes and immune cells. The results of the immune cell correlation analysis show that the expression level of the genes was most closely related to macrophages. The expression levels of BCHE、MAMDC2 and PLCXD3 were moderately positively correlated with the degree of macrophage infiltration while the expression levels of AGT、GAMT、CDH19、ADCYAP1R1、RXRG and UBE2QL1 were weakly positively correlated with the degree of macrophage infiltration (see **Figure 7** for details).

### Correlation Analysis of Sample Sequencing

**Figure 8:**
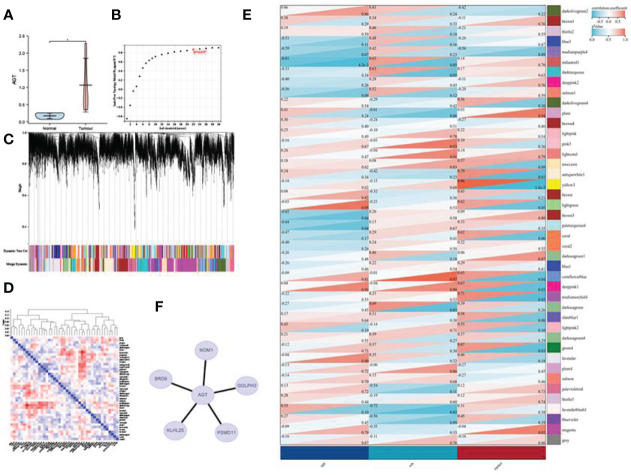
WGCNA analysis: **(A)** Expression of AGT in gastric cancer based on the sequencing expression matrix; **(B)** soft threshold; **(C)** gene clustering; **(D)** module eigenvector clustering; **(E)** module and phenotype correlation heat map; **(F)** AGT coexpression network. *p < 0.05.

After expression analysis and the observation of the expression in the stages of gastric cancer, we found that AGT and NrCAM were up-regulated in gastric cancer and that their appearance could increase with the increase of the stage. Moreover, the predictive results of these two genes in gastric cancer show that the higher their expression, the worse the prognosis. Therefore, we collected gastric cancer tissue samples for transcriptome analysis to further observe the expression of AGT and NRCAM in gastric cancer. The results reveal that only AGT had significant expression differences in gastric cancer tissues, and the up-regulated expression was consistent with the above analysis results based on TCGA. Next, we performed WGCNA analysis based on the expression matrix obtained by sequencing, identified the module where AGT is located (antiquewhite1), and explored the genes that have a coexpression relationship with AGT. The results show that BRD9,GOLPH3, NOM1, KLHL25, and PSMD11 interacted with AGT (see **Figure 8** for details).

## Discussion

Like other solid tumors, gastric cancer comprises several molecular subtypes with diverse biological characteristics. The increasing understanding of molecular pathways provides a basis for innovative therapies (10). Molecular targeted therapy can be used to identify specific oncogenic targets of tumor cells, killing tumor cells (11). In addition, the therapy has been shown to regulate the immune response, as some molecularly targeted drugs can increase tumor antigen expression and promote the antigen presentation of antigen-presenting cells to induce a more robust antitumor immune response (12). The expression of mRNA, a transient intermediate between genes and proteins, has shown therapeutic potential in various applications, including viral vaccines, protein replacement therapy, cancer immunotherapy, cell reprogramming, and genome editing (13). Therefore, mRNA has become a new therapeutic agent for preventing and treating various diseases (14). Using dynamic trend analysis, differential analysis, and predictive analysis, this study screened 10 genes with prognostic significance in gastric cancer (RXRG, AGT, BCHE, UBE2QL1, PLCXD3, ADCYAP1R1, NRCAM, MAMDC2, CDH19, and GAMT). Expression analysis and stage expression analysis also showed that they had high or low expression in gastric cancer and changed dynamically with the evolution of the gastric cancer stage.

The immune microenvironment plays an essential role in the development of digestive system cancer. Tumor infiltrating immune cells play a crucial role in suppressing or supporting tumor growth and development and can be effectively targeted by drugs, associated with patient survival time (15). Other studies have shown that macrophages induce PD-L1 expression and help gastric cancer cells escape cytotoxic T cell death. In addition, they can promote the proliferation of gastric cancer cells by regulating the expression of PD-L1 (16).

Moreover, macrophage infiltration is significantly correlated with the prognosis of patients with gastric cancer (17). ADCYAP1R1 has anti-inflammatory, neuroprotective and regenerative properties, and its expression may also be influenced by immune/inflammatory stimuli (18). It has been shown that deletion of ADCYAP1R1 in naïve mice results in the absence of retinal ganglion neurons and their dendrites, while increased axonal pathology and secondary increases in microglia/macrophages are also evident in the optic nerve (19). CDH19 is thought to be a member of calmodulin and establishes and maintains intercellular junctions (20). Some studies have shown that CDH19 may be a potential candidate as an immunotherapeutic target for breast cancer patients (21). MAMDC2 is a proteoglycan of completely unknown function, containing five MAM structural domains. They mediate interactions and stability and can act as an adhesion structural domain for surface receptors (22). Some studies have also shown that MAMDC2 plays a key role in the development of invasive ductal carcinoma of the breast (23). Although no clear studies have shown their direct role with immune cells in tumors, they also show that they play an important role in tumors. We found that the expression levels of BCHE, MAMDC2 and PLCXD3 were moderately positively correlated with the degree of macrophage infiltration while the expression levels of AGT, GAMT, CDH19, ADCYAP1R1, RXRG and UBE2QL1 were weakly positively correlated with the degree of macrophage infiltration. These results show that these genes can potentially regulate the recruitment and activation of immune cells in gastric cancer.

This study found that AGT is highly expressed in gastric cancer and up-regulated with the increase of the stage. This continuous high expression may also be related to the poor prognosis of patients. The results based on sample sequencing also show that the expression of AGT was up-regulated in gastric cancer as compared with the standard group. AGT is an inactive precursor of potent vasoactivity and the salt-retention hormone angiotensin II, and an elevated level of it is the primary precursor in the pathogenesis of hypertension (24, 25). AGT also plays a critical role in tumors. Oncogenic effects of high glucose on breast cancer cell growth and metastasis (26). Studies have shown that AGT can also inhibit the invasion and migration of colorectal cancer (27). In this study, we performed a WGCNA of the sequenced expression matrix, which revealed that AGT was located in the antiquewhite1 module, closely related to gastric cancer. Based on the genes of this module, we explored the genes that have a coexpression relationship with AGT. The analysis shows that BRD9, GOLPH3, NOM1, KLHL25, and PSMD11 interact with AGT.

In conclusion, the expression of AGT is up-regulated in gastric cancer and the gastric cancer stages, and its continuous up-regulation is closely related to the poor prognosis of patients with gastric cancer. It is suggested that AGT may contribute to gastric cancer progression and could be used as a potential biomarker or therapeutic target in gastric cancer. However, there are deficiencies in this study, and *in vitro* experiments are needed to determine the critical signal pathways related to AGT.

## Data Availability Statement

The original contributions presented in the study are included in the article/supplementary material. Further inquiries can be directed to the corresponding author.

## Author Contributions

All authors listed have made a substantial, direct, and intellectual contribution to the work and approved it for publication.

## Funding

This study was supported by the key discipline “minimally invasive oncology of acupuncture and Moxibustion” (2017-XK-A12) of Zhejiang Administration of traditional Chinese medicine and the inheritance studio of Pangde Xiang famous old traditional Chinese medicine experts (2A12012014).

## Conflict of Interest

The authors declare that the research was conducted in the absence of any commercial or financial relationships that could be construed as a potential conflict of interest.

## Publisher’s Note

All claims expressed in this article are solely those of the authors and do not necessarily represent those of their affiliated organizations, or those of the publisher, the editors and the reviewers. Any product that may be evaluated in this article, or claim that may be made by its manufacturer, is not guaranteed or endorsed by the publisher.
